# Impact of adverse reactions to first-generation antipsychotics on treatment adherence in outpatients with schizophrenia: a cross-sectional study

**DOI:** 10.1186/s12991-021-00348-0

**Published:** 2021-04-24

**Authors:** Merhawi Bahta, Azieb Ogbaghebriel, Mulugeta Russom, Eyasu H. Tesfamariam, Tzeggai Berhe

**Affiliations:** 1 School of Pharmacy, Asmara College of Health Sciences, Asmara, Eritrea; 2 Department of Pharmaceutical Sciences, College of Pharmacy, Princess Nourah Bint Abdulrahman University, Riyadh, Kingdom of Saudi Arabia; 3 Eritrean Pharmacovigilance Centre, National Medicines and Food Administration, Asmara, Eritrea; 4 Department of Statistics, College of Science, Mainefhi, Eritrea; 5 Department of Addictions and Mental Health, University of Alberta, Edmonton, Canada; 6 Present Address: Department of Psychiatry, Faculty of Medicine & Dentistry, University of Alberta, Edmonton, Canada

**Keywords:** Schizophrenia, First-generation antipsychotics, Adverse drug reactions, Treatment adherence

## Abstract

**Background:**

Antipsychotics are well-known to cause potentially serious and life-threatening adverse drug reactions (ADRs) that have been reported to be also one of the major reasons for non-adherence. In Eritrea, shortage of psychiatrists and physicians, inadequacy of laboratory setups and unavailability of second-generation antipsychotics in the national list of medicines would seem to amplify the problem. This study’s objective is to determine the impact of adverse effects of first-generation antipsychotics on treatment adherence in outpatients with schizophrenia at Saint Mary Neuro-Psychiatric National Referral Hospital.

**Methods:**

A cross-sectional study design was employed. All eligible adult patients with diagnosed schizophrenia (*n* = 242) who visited the hospital during the study period were enrolled. Data on ADRs, adherence and other variables were collected from patients using a self-administered questionnaire, interview and through medical cards review. The collected variables were analyzed using SPSS 22.0 with descriptive and multivariable logistic regression analysis. Statistical significance was tested at *p* value < 0.05.

**Results:**

Greater than one-third (35.5%) of the patients with schizophrenia were non-adherent to treatment. The odds of non-adherence increased 1.06 times for each unit increase in the total ADR score (AOR = 1.06, 95% CI 1.04, 1.09). Patients with extrapyramidal (AOR = 44.69, 95% CI 5.98, 334.30), psychic (AOR = 14.90, 95% CI 1.90, 116.86), hormonal (AOR = 2.60, 95% CI 1.41, 4.80), autonomic (AOR = 3.23, 95% CI 1.37, 7.57) and miscellaneous reactions (AOR = 2.16, 95% CI 1.13, 4.13) were more likely to be non-adherent compared to their counterparts.

**Conclusion:**

Poor treatment adherence was found to be substantial which was attributed to total ADR score, extrapyramidal, hormonal, psychic, autonomic and miscellaneous categories of reactions of the LUNSERS. To improve treatment adherence, early detection and management of adverse effects and inclusion of second-generation antipsychotics are recommended.

**Supplementary Information:**

The online version contains supplementary material available at 10.1186/s12991-021-00348-0.

## Background

Schizophrenia is a mental illness characterized by heterogeneous combinations of symptoms, which include, according to DSM V delusions, hallucinations, disorganized speech and behavior and negative symptoms. Schizophrenia can result in pervasive decline of personal, social and occupational functioning of the individual [1]. Antipsychotic medications are one of the primary approaches in the management of schizophrenia and other psychotic disorders [2, 3] in which adherence to these drugs has paramount importance in achieving the therapeutic outcome.

Adherence is defined as the degree to which a patient follows medical advice, prescriptions and therapeutic recommendations from a healthcare provider [4]. Non-adherence to antipsychotic medications is common but results are inconsistent across studies. A review article reported a mean non-adherence rate of 40.5% (range: 4–72%) [5]. A study conducted among insightful patients diagnosed with schizophrenia in Eritrea also reported 33% non-adherence to antipsychotics [6].

Medication adherence is influenced by several factors, that include the World Health Organization’s five dimensions: Socio-economic situation, health system, condition, patient, and therapy [7]. Besides, several studies have mainly attributed psychiatric treatment non-adherence to the experience of antipsychotic-induced ADRs [8–22]. Ultimately, non-adherence to antipsychotic medications often leads to relapse in psychotic disorders, repeated hospitalizations, increased risk of suicide [23] and higher treatment costs [10]. In our previously published study, which utilized the same study population as the current one, with the aim of determining the magnitude, nature, and ADR risk factors of first-generation antipsychotics, we found psychic, autonomic, extrapyramidal, hormonal and miscellaneous ADRs to be highly prevalent [24]. This study was, therefore, aimed at determining the level of adherence and impact of adverse effects of first-generation antipsychotics on treatment adherence in outpatients with schizophrenia at Saint Mary Neuro-Psychiatric National Referral Hospital (SMNPNRH) in Asmara—Eritrea.

## Materials and methods

The detailed methodological approach used in this study was published elsewhere in the previous study [24]. In brief, it was a cross-sectional study carried out at the outpatient department of the SMNPNRH in Asmara, Eritrea. Schizophrenia diagnosed patients with no co-morbidities (any additional chronic diseases for which patients may take other medications), aged 18 years and above, attending the National Referral Hospital between August 28 and October 12, 2018 (*n* = 242) were enrolled into the study. Those who were exposed to antipsychotic medications, mainly to one or more of the following FGAs: chlorpromazine tablet, fluphenazine decanoate intramuscular injection, and haloperidol tablet for at least 1 month prior to the commencement of the study and clinically stable enough to communicate and willing to participate were included in the study. After informed consent was obtained, data on ADRs and adherence was collected from patients using a self-administered questionnaire, while socio-demographic and clinical data were collected using interview and clinical card review. Doses of the antipsychotics were retrieved from the clinical card and was converted to chlorpromazine equivalents (mg/day) to allow for dose comparison across the different antipsychotics based on conversion factors obtained from the literature [25].

Validated and reliable tools, the Liverpool University Neuroleptic Side-Effect Rating Scale (LUNSERS) [26] and Medication Adherence Rating Scale (MARS) [27, 28] were used to evaluate adverse effects and treatment adherence, respectively. Respondents self-rated how much they experienced the adverse effects in the last month using the 41 items of the LUNERS. The 41 ADRs are grouped into seven categories, namely: extra-pyramidal (restlessness, muscle stiffness, parts of body moving on their own, shakiness, slowing of movements, muscle spasms, over-wet or drooling mouth), anti-cholinergic (blurred vision, passing a lot of water, constipation, dry mouth, difficulty passing water), other autonomic (feeling sick, dizziness, increased sweating, palpitations, diarrhea), allergic (sensitivity to sun (photosensitivity), itchy skin, rash, new or unusual skin marks), psychic (tiredness, tension, depression, difficulty getting to sleep, difficulty staying awake during the day, sleeping too much, difficulty in concentrating, difficulty in remembering things, increased dreaming, lack of emotions), hormonal (reduced sex drive, difficulty achieving climax, increased sex drive, period problems, periods less frequent, swollen or tender chest) and miscellaneous (headaches, putting on weight, losing weight, pins and needles) reactions. Besides, the LUNSERS included 10 red herrings, symptoms that do not directly relate to known antipsychotic ADRs.

The primary outcome of this study was impact of adverse effects on treatment adherence. Descriptive analyses of the demographic, clinical, LUNSERS items, and MARS items were performed using frequency (percent) for categorical variables as well as mean (SD) and median (IQR) for continuous variables. The prevalence of ADRs was determined by the percentage of patients who scored one or more on the relevant LUNSERS items or subscales. Moreover, the means of the total ADRs and red herring score of each client were also calculated by summing the values on all of the items. Total ADRs score implies the degree of intensity of ADRs that ranged from 0–164, with higher scores reflecting greater number and perceived severity of ADRs. Mean red herring score indicates the accuracy of the self-report that ranged from 0–40, with a score less than 20 reflecting higher accuracy of the self-report. Rate of non-adherence was calculated based on the MARS score, where a score of six or higher was considered to be adherent. To assess the association of non-adherence with each demographic and clinical variable, as well as red herrings, a bivariate logistic regression was used. Then, multivariable logistic regression was employed to assess the association of non-adherence and adverse effects after controlling the confounding effect of the significant variables at bivariate level. Crude and adjusted odds ratio (with 95% confidence interval) were computed and reported. *p* values less than 0.05 were considered as significant throughout the study.

## Results

Details of the socio-demographic and clinical characteristics of the study participants were reported in our previously published article aimed to determine the magnitude, nature and risk factors associated with first-generation antipsychotics [24]. In summary, the study participants were 242 patients (response rate = 96.4%) with diagnosed schizophrenia attending outpatient department of the SMNPNRH. The mean age and mean body weight of the respondents were 39.73 years (SD = 11.22, range = 18–70) and 61.47 kg (SD = 11.56, range = 35–100), respectively. The majority of the study participants (67%) were residents of Asmara. About 20% were current smokers and 10% reported that they regularly consume alcohol. All were taking at least one FGA with a mean duration of illness of 157.90 months (~ 13 years). Concurrent use of two or more FGAs was in about half (49.2%) of the study participants. Besides, majority (46.7%) of the participants used a combination of oral and long acting injectable followed by oral only (32.2%) and long acting injectable only (21.1%).

### Adherence level

Based on the MARS score, greater than one-third (*n* = 86, 35.5%: 95% CI 29.5, 41.9) of the patients with schizophrenia were non-adherent to their treatment. The mean MARS score of the participants was 6.7 (SD = 2.89, minimum = 0 and maximum = 10). According to the item wise percentage distribution of MARS scale, more than half of the participants (57.4%) reported that they felt tired and sluggish with the medication and 46.7% reported that they felt weird, like being zombie, following the intake of the antipsychotics (Additional file 1).

### Adherence and its association with ADRs

Bivariate logistic regression was performed to assess the impact of each demographic and clinical variable along with red herrings score on the likelihood that respondents would report non-adherence, as shown in Table 1. Secondary school (OR = 2.48, 95% CI 1.04, 5.90) levels were more likely to be non-adherent than those with primary/no formal education. Self-reported non-adherence was also highly reported in smokers compared to the non-smokers (OR = 2.13, 95% CI 1.12, 4.04). Similarly, the odds of non-adherence increased 1.002 times for each unit increase in the antipsychotic dose (OR = 1.002, 95% CI 1.0001, 1.0037), while the odds of non-adherence increased 1.098 times for each unit increase in the red herrings score (OR = 1.098, 95% CI 1.0073, 1.1975) (Table 1).

**Table 1:** Demographic and clinical variables as predictors of non-adherence at bivariate level

Variables	Adherent		COR (95% CI)	*p *value
	Yes	No		
	n (%)	n (%)		
Gender				
Male	89 (57.1)	49 (57.0)	1.00 (0.59, 1.70)	0.991
Female	67 (42.9)	37 (43.0)	Ref	
Educational level				
Middle	30 (19.2)	24 (27.9)	3.00 (1.16, 7.73)	0.023^a^
Secondary	68 (43.6)	45 (52.3)	2.48 (1.04, 5.90)	0.040^a^
Higher	28 (17.9)	9 (10.5)	1.25 (0.41, 3.56)	0.735
Primary/no formal education	30 (19.2)	8 (9.3)	Ref	
Employment				
Unemployed	114 (73.1)	55 (64)	0.65 (0.37, 1.15)	0.140
Employed	42 (26.9)	31 (36)	Ref	
Marital status				
Single	67 (42.9)	43 (50)	2.09 (0.85, 5.03)	0.102
Married	63 (404)	35 (40.7)	1.01 (0.74, 4.14)	0.195
Divorced/widowed/separated	26 (16.7)	8 (9.3)	Ref	
Residence				
Asmara	100 (64.1)	62 (72.1)	1.45 (0.81, 2.57)	0.207
Out of Asmara	56 (35.9)	24 (27.9)	Ref	
Smoking				
Yes	24 (15.4)	24 (27.9)	2.13 (1.12, 4.04)	0.021^a^
No	132 (84.6)	62 (72.1)	Ref	
Alcohol				
Yes	12 (7.7)	13 (15.1)	2.14 (0.93, 4.92)	0.074
No	144 (92.3)	73 (84.9)	Ref	
Number of APs				
Single	84 (53.8)	39 (45.3)	0.71 (0.42, 1.21)	0.206
Multiple	72 (46.2)	47 (54.7)	Ref	
ADRs-Counseling				
Yes	81 (51.9)	41 (47.7)	0.84 (0.50, 1.43)	0.527
No	75 (48.1)	45 (52.3)	Ref	
Drug formulation				
Long acting injectable	38 (24.4)	13 (15.1)	0.93 (0.51, 1.68)	0.806
Oral and long acting injectable	69 (44.2)	44 (51.2)	0.54 (0.26,1.12)	0.097
Oral	49 (31.4)	29 (33.7)	Ref	

Variables	Adherent		COR (95% CI)	*p *value
	Yes	No		
	Md (IQR)	Md (IQR)		
Age	39.0 (16.0)	19.0 (15.0)	0.990 (0.9668,1.0139)	0.412
Weight	60.0 (18.0)	60.0 (12.0)	0.004 (0.9809, 1.0267)	0.758
Duration of illness	135.5 (144.0)	159.0 (163.3)	1.000 (0.9981, 1.0027)	0.734
Mean antipsychotic dose	196.2 (196.2)	237.5 (226.4)	1.002 (1.0001, 1.0037)	0.034^a^
Red herrings score	0.0 (2.0)	0.0 (4.0)	1.098 (1.0073, 1.1975)	0.033^a^

*COR*  Crude odds ratio, *CI* confidence interval, *Md* median, *IQR* inter quartile range, *n* number of participants

^a^Significant

Multivariable logistic regression was also performed to assess the impact of the total ADRs score and each ADRs sub scale on the likelihood that the patients would report non-adherence after controlling the effect of dose, smoking, educational level and red herrings as they were significant at bivariate level (Table 2). The odds of non-adherence increased 1.06 times for unit increase in the total ADR scale’s score (AOR = 1.06, 95% CI 1.04, 1.09). All ADRs subscales except anticholinergic and allergic were also statistically significantly associated with the likelihood of non-adherence. Patients with extrapyramidal (AOR = 44.69, 95% CI 5.98, 334.30), psychic (AOR = 14.90, 95% CI 1.90, 116.86), hormonal (AOR = 2.60, 95% CI 1.41, 4.80), autonomic (AOR = 3.23, 95% CI 1.37, 7.57) and miscellaneous reactions (AOR = 2.16, 95% CI 1.13, 4.13) were more likely to be non-adherent compared to their counterparts (Table 2).

**Table 2:** Adverse drug reactions as predictors of non-adherence at multivariable level

Variables	Adherent		AOR (95% CI)	*p* value
	Yes	No		
	*n* (%)	*n* (%)		
Extrapyramidal ADRs				
Yes	101 (64.7)	85 (98.8)	44.69 (5.98, 334.30)	0.000^a^
No	55 (35.3)	1 (1.2)	Ref	
Psychic ADRs				
Yes	136 (87.2)	85 (98.8)	14.90 (1.90, 116.86)	0.010^a^
No	20 (12.8)	1 (1.2)	Ref	
Allergic ADRs				
Yes	59 (37.8)	48 (55.8)	1.70 (0.92, 3.14)	0.090
No	97 (62.2)	38 (44.2)	Ref	
Anticholinergic ADRs				
Yes	109 (69.9)	68 (79.1)	1.29 (0.65, 2.52)	0.467
No	47 (30.1)	18 (20.9)	Ref	
Hormonal ADRs				
Yes	76 (48.7)	65 (75.6)	2.60 (1.41, 4.80)	0.002^a^
No	80 (51.3)	21 (24.4)	Ref	
Autonomic ADRs				
Yes	111(71.2)	78 (90.7)	3.23 (1.37, 7.57)	0.007^a^
No	45 (28.8)	8 (9.3)	Ref	
Miscellaneous ADRs				
Yes	94 (60.3)	67 (77.9)	2.16 (1.13, 4.12)	0.020^a^
No	62 (39.7)	19 (22.1)	Ref	

Variables	Adherent		AOR (95% CI)	*p* value
	Yes	No		
	Md (IQR)	Md (IQR)		
Total ADRs score	20.0 (20.75)	38.0 (26.25)	1.06 (1.04, 1.09)	0.000^a^

Educational level, dose, smoking and red herrings were controlled in the relationship of total ADRs score and each LUNSERS subscale with non-adherence

*ADR* Adverse drug reaction, *AOR* adjusted odds ratio, *CI* confidence interval, *n* number of participants, *Md* median, *IQR* inter quartile range;

^a^Significant

## Discussion

In this study, one in three of the study participants were found to be non-adherent to their treatment regimens. This is more or less similar to a finding reported in a review of similar studies (40.5%) published in 2002 [5] and other latest studies conducted elsewhere [6, 15, 29] but lower than some studies reported in Ethiopia (48.4%) [22], Nigeria (51.7%) [16], Kenya (60.4) [21] and Egypt (74%) [30]. On the other hand, lower rates of non-adherence than the present finding were reported in Latin American community-dwelling (19.8%) [31] and northern Ethiopia (26.5%) [17]. The variations in rates of non-adherence among the studies could be explained by the differences in the methods of assessment of medication adherence and different cut-off values. Besides, it could also be attributed to some unique nature of the study populations and quality of mental health services in the study sites. Moreover, the chlorpromazine dose equivalent in our study population was about 245 mg/d on average, which is considered low. According to a Cochrane review, doses of less than 400 mg/d (low dose) and those of intermediate dose of 400–800 mg/d have similar efficacy with the higher dose obviously producing more adverse effects [32]. Therefore, the dose-related adverse effect severity in our study could have probably been lower and hence contributing to the more favorable adherence rate than what has been reported in other African studies [16, 21, 22, 30].

In the current study, total ADRs score which implies the accumulated number and severity of adverse effects was significantly associated with treatment non-adherence. The adverse effect categories of extrapyramidal, psychic, hormonal, autonomic and miscellaneous which were found to be highly prevalent (58.3 to 91.3%) in this study population [24] were also found to be significant determinants of non-adherence. This could be due to the fact that the EPS including restlessness, muscle stiffness, slowing of movements and muscle spasms could interfere with the daily living activities and can lead to reduced quality of life which make the patient to stand out as different, hence contributing to stigma and non-adherence. Besides, psychic adverse effects can result in decreased cognitive functioning which can affect the quality of life and adherence negatively. Moreover, the miscellaneous reactions such as weight gain could lead to subjective dissatisfaction about their appearance, while the hormonal adverse effects such as decreased sexual functioning may potentially affect their relationship with their partners resulting to stigma and non-adherence.

Association of specific adverse effect categories and non-adherence found in the present study is in line with some studies, which reported extrapyramidal [9, 15, 21, 22], hormonal [8, 9, 15], psychic [8, 9, 15, 18], and miscellaneous reactions such as weight gain [9] as determinants of non-adherence. Nevertheless, some studies reported a minimal or no relationship between antipsychotic ADRs and risk of non-adherence [33, 34]. The study done by Terence et.al 2008 [33], reported no significant association of adherence and all of the seven LUNSERS subscales.

These findings imply that any ADR can be an important risk factor for non-adherence to treatment. However, it can also be influenced by the patients' levels of insight and ability to weigh up the benefits and risks of prescribed medications [35] and their experience with mental health services. Therefore, when patients are required to take antipsychotic medications a patient-centered approach with culture-sensitive psychoeducational programs emphasizing the patients' treatment objectives toward improving psychosocial functioning, quality of life, and recovery and enhancing relationships between mental health professional and patients and their communities should be a major part of mental health service development plan [36]. Moreover, early detection and management of ADRs have utmost importance in improving treatment adherence.

In our study hospital, the challenges of non-adherence appear to encompass more than ADRs. In addition to including second-generation antipsychotics into the National List of Medicines, the need to plan for appropriate staffing in the facility, to introduce community based mental health, rehabilitation and counseling programs is recognized. A compartmental pill box to help patients and caregivers to keep truck of their medications might also be helpful.

The use of validated and reliable scales to measure treatment adherence and ADRs was the main strength of this study. Besides, the accuracy of the patients’ self-report of ADRs was ascertained by the red herrings score of less than 20. However, this study had several limitations. In some instances, there might be exposure and outcome misclassification bias as it could be challenging to differentiate if the ADR was drug or condition related. This could have been assessed using treatment outcome tools, such as the Positive and Negative Syndrome Scale (PANSS) [37], which was not done in this study. The self-reported treatment adherence might also lead to information bias, possibly resulting in inaccurate estimates of the reported adherence. Besides, the selection of stable patients with schizophrenia attending OPD without co-morbidities or concomitant drug use during the study period might have excluded patients with severe or early onset ADRs and possibly higher risk for non-adherence. Moreover, the chlorpromazine-equivalent dose is based on dopaminergic activity, and not taking possible positive or negative synergic multi-receptor effects on treatment outcomes into account was the study's limitation.

## Conclusion

Poor treatment adherence to first-generation antipsychotics was found to be substantial. Total ADR score, extrapyramidal, hormonal, psychic, autonomic and miscellaneous categories of reactions of the LUNSERS were identified as risk factors for the poor treatment adherence. To improve treatment adherence, early detection and treatment of adverse effects, appropriate patient counseling on ADRs, improving staffing, and inclusion of second-generation antipsychotics are recommended.

## Supplementary Information

**Additional file 1. **Item wise percentage distribution of MARS scale. *n* = number of participants.

## Data Availability

The datasets used and analyzed during the current study are available from the corresponding author on request.

## Abbreviations

ADRs: Adverse drug reactions
FGAs: First-generation antipsychotics
LUNSERS: Liverpool university neuroleptic side-effect rating scale
MARS: Medication adherence rating scale
SMNPNRH: Saint mary neuro psychiatric national referral hospital
