# Supplementary material for: Impact of adverse reactions to first-generation antipsychotics on treatment adherence in outpatients with schizophrenia: a cross-sectional study
Source: Ann Gen Psychiatry. 2021 Apr 24;20:27. doi: 10.1186/s12991-021-00348-0 (PMC8070289; doi:10.1186/s12991-021-00348-0)
Supplement: Supplementary file 1 — Additional file 1. Item wise percentage distribution of MARS scale. n = number of participants. [file 12991_2021_348_MOESM1_ESM.docx]

**S 1: Item wise percentage distribution of MARS scale**

n= number of participants

|  | Response | |
| --- | --- | --- |
| MARS items | Yes  n (%) | No  n (%) |
| 1. Do you ever forget taking medication? | 94 (38.8) | 148 (61.2) |
| 2. Are you careless at times about taking your medication? | 84 (34.7) | 158 (65.3) |
| 3. When you feel better, do you sometimes stop taking your medication? | 68 (28.1) | 174 (71.9) |
| 4. Sometimes when you feel worse when you take medicine, do you stop taking it? | 67 (27.7) | 175 (72.3) |
| 5. I take my medication only when I am sick. | 26 (10.7) | 216 (89.3) |
| 6. It is unnatural for my mind and body to be controlled by medications. | 74 (38.6) | 168 (69.4) |
| 7. My thoughts are cleared on medication. | 162 (66.9) | 80 (33.1) |
| 8. By staying on medication, I can prevent getting sick. | 188 (77.7) | 54 (22.3) |
| 9. I feel weird, like a zombie on medication. | 113 (46.7) | 129 (53.3) |
| 10. Medication makes me feel tired and sluggish. | 139 (57.4) | 103 (42.6) |
